# Interpolation time-optimized aortic pulse wave velocity estimation by 4D flow MRI

**DOI:** 10.1038/s41598-023-43799-z

**Published:** 2023-09-30

**Authors:** Sungho Park, Minseong Kwon, Hyojin Nam, Hyungkyu Huh

**Affiliations:** 1 Daegu-Gyeongbuk Medical Innovation Foundation, Medical Device Development Center, Daegu, 41061 South Korea; 2 Institute of Medical Devices, Kangwon National University, Chuncheon, South Korea; 3 Department of Radiology, Section of Pediatric Radiology, Children’s Hospital Colorado, University of Colorado Anschutz Medical Campus, Aurora, CO USA

**Keywords:** Biomedical engineering, Magnetic resonance imaging

## Abstract

Four-dimensional flow magnetic resonance imaging-based pulse wave velocity (4D flow PWV) estimation is a promising tool for measuring regional aortic stiffness for non-invasive cardiovascular disease screening. However, the effect of variations in the shape of flow waveforms on 4D flow PWV measurements remains unclear. In this study, 4D flow PWV values were compared using cross-correlation algorithm with different interpolation times (iTs) based on flow rate and beat frequency. A critical iT (iT_Crit_) was proposed from in vitro study using flexible and stiff phantom models to simultaneously achieve a low difference and a low computation time. In vivo 4D flow PWV values from six healthy volunteers were also compared between iT_Crit_ and the conventionally used interpolation time of 1 ms (iT_1 ms_). The results indicated that iT_Crit_ reduced the mean difference of in vitro 4D flow PWV values by 19%, compared to iT_1 ms_. In addition, iT_Crit_ measured in vivo 4D flow PWV, showing differences similar to those obtained with iT_1 ms_. A difference estimation model was proposed to retrospectively estimate potential differences of 4D flow PWV using known values of PWV and the used iT. This study would be helpful for understanding the differences of PWV generated by physiological changes and time step of obtained flow waveforms.

## Introduction

Aortic stiffening is a determinant predictor of cardiovascular disease, which is a leading cause of morbidity and mortality worldwide^1,2^. Previous studies have demonstrated the clinical importance of aortic stiffness, which has been associated with diabetes^3^, left ventricular remodeling^4,5^, and cerebral small vessel disease^6^. Aortic stiffness is commonly assessed by the hemodynamic biomarker of pulse wave velocity (PWV)^7^. PWV is calculated as the traveling distance of pressure or flow waveforms between two anatomical locations, divided by the pulse transit time (PTT) taken during the traveling distance.

A gold standard of PWV estimation is catheter-based pressure measurements by directly calculating the temporal shift and the travel distance of traveling pressure waves^8^. However, the catheter-based modalities are rarely used because of their invasiveness, which has prompted the development of non-invasive PWV estimations. Phase contrast (PC) magnetic resonance imaging (MRI) becomes a promising tool, which non-invasively evaluates PWV by using velocity-derived traveling flow waveforms at two locations. 2D PC MRI accurately measures aortic arch PWV with a very high temporal resolution^9^. In addition, 2D PC MRI provides regional PWV data along an aortic arch, which is not available in carotid-to-femoral PWV estimation by tonometry or Doppler ultrasound^10,11^. The clinical applicability of 2D PC MRI-based PWV measurement has been validated with ultrasound measurement using in vitro phantom models, as well as exhibiting a significant difference between healthy and elderly subjects^12^. However, while 2D PC MRI is capable of measuring regional PWV especially for an arch, there remains a concern in simultaneously estimating both global and regional aortic PWV as aortic stiffness varies regionally along an entire vessel^13^. This may have further implications for focal diseases including thoracic or abdominal aortic aneurysms^14^ and atherosclerosis^15^.

Recently, some researchers have explored PWV estimation by using 4D flow MRI, which provides time-resolved and 3D volumetric velocity information of blood flow along the entire vessel^16,17^. An exact distance can be obtained from vessel anatomy through PC magnetic resonance angiography (MRA). With the known distance, 4D flow MRI enables regional PWV assessment regardless of vascular geometry by extracting cross-sectional image planes at any locations along the entire vessel. In addition, retrospective data processing of blood flow waveforms obtained from image planes eliminates the need for expertise required for real time PWV estimation. With these advantages, 4D flow MRI-derived PWV (4D flow PWV) has been utilized to identify aortic stiffening in stroke patients with aortic atherosclerosis^15,18,19^, Alzheimer’s disease^20^, and with tricuspid aortic valve and degenerative aneurysm^21^. However, 4D flow PWV significantly varies according to the adopting algorithms, such as time to peak, time to foot (TTF), cross-correlation, wavelets, and Fourier transforms^19,22^, even when using the same flow waveforms. Previous attempts have shown that cross-correlation is the most reliable^23,24^, however, these approaches have only focused on the comparisons of differences in 4D flow PWV among existing algorithms or other modalities. However, we further hypothesize that differences in the shape of flow waveforms will also affect 4D flow PWV measurement, even when using a same algorithm.

Herein, we exploit both in vitro and in vivo 4D flow MRI to identify differences in 4D flow PWV using the cross-correlation algorithm. The shape of flow waveforms was modulated as a function of interpolation time (iT), flow rate, and beat frequency. The effects of these parameters were also compared with flexible and stiff in vitro phantom models. A critical iT (iT_Crit_) was proposed to simultaneously achieve a low difference and a low computation time using the coefficient of difference. In vivo 4D flow PWV values were subsequently measured in 6 healthy subjects. A difference estimation model was developed by using iT_Crit_ normalized by distance according to PWV obtained from in vitro and in vivo data. This estimation model will allow us to provide a potential difference of 4D flow PWV using known values of PWV and the used iT. This study provides the evidence that potential difference may occur in traditional 4D flow PWV measurement especially in a patient with high PWV.

## Methods

### In vitro data acquisition

**Figure 1:**
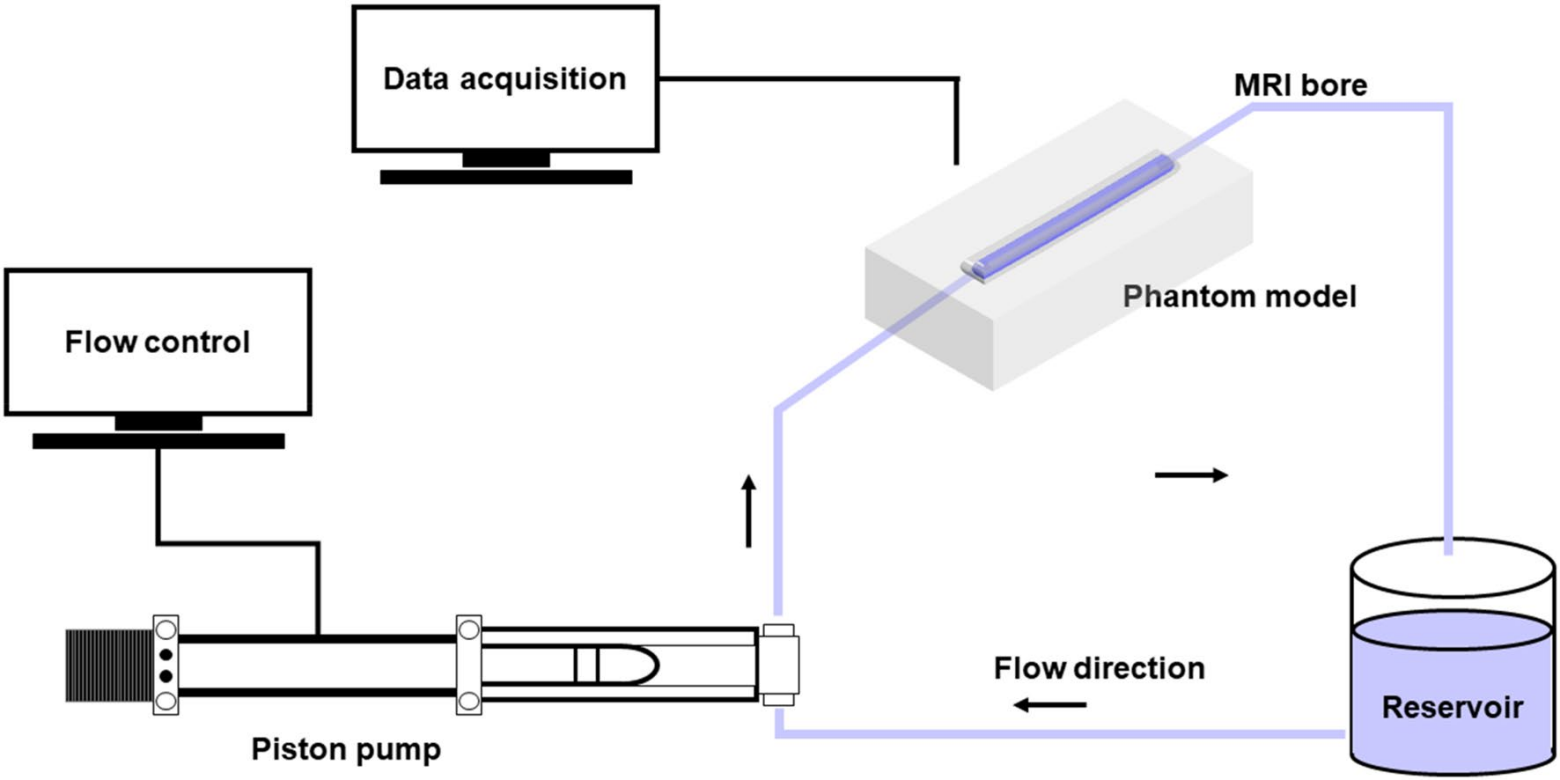
Experimental setup for in vitro 4D flow PWV measurement.

Flexible and stiff silicon phantom models (P_Flex_ and P_Stiff_) were used for measuring in vitro 4D flow PWV. P_Flex_ and P_Stiff_ have a length of 30 cm, inner diameter of 22 mm, and outer diameters of 26 mm and 30 mm, respectively. Beat frequency and stroke volume were simulated using an in-house piston pump to simulate blood flow waveform of the human aorta^25^. Inlet flow parameters were modulated using LabVIEW software (National Instruments, Austin, TX, USA). Flow rates were named as Q_Low_, Q_Mid_, and Q_High_ corresponding to Reynolds number (Re = $\rho QD/\mu A$) of 1386, 2080, and 3466, respectively (Table 1), where $\rho$ is the density, Q is the flow rate, D is the diameter, $\mu$ is the dynamic viscosity, and A is the area. In addition, beat frequencies of 60, 80, 100, and 120 beats per minute (BPM) were summarized with Womersley number (Wo = $D/2\sqrt{\rho 2\pi f/\mu }$) in Table 2, where f is the frequency. Re and Wo numbers were similarly matched with those observed in the human aorta, which corresponds to the ranges of 900–4600 and 12‒16, respectively^26^. However, Wo number seems to increase during the alignment of beating frequency to a reasonable range observed in human. 4D flow MRI measurements were performed using a commercial 3-T MRI scanner (Skyra, Siemens AG, Munich, Germany). The three-directional encoding velocity (Venc) varies from 100 to 280 cm s^−1^ depending on flow rate, beat frequency, and phantom model, and the scan parameters were as follows: echo time = 2.5‒3 ms, repetition time = 40‒50 ms, temporal resolution = 40.0‒45.3 ms, flip angle = 15°, field of view = 280 × 320 × 50 mm^3^, voxel size = 2 × 2 × 2 mm^3^. The total acquisition time was approximately 10 min. The experimental setup of in vitro 4D flow PWV measurement is illustrated in Fig. 1.

**Table 1:** Flow rate conditions and Reynolds number for in vitro PWV estimation.

	Q_Low_	Q_Mid_	Q_High_
Flow rate (mL min^−1^)	1442	2164	3606
Reynolds number	1386	2080	3466

**Table 2:** Womersley number for in vitro PWV estimation.

Beat frequency (BPM)				
	60	80	100	120
Womersley number	27.53	31.78	35.54	38.93

### In vivo data acquisition

In vivo human study was conducted after obtaining approval from the Institutional Review Board (IRB) of the Daegu Gyeongbuk Medical Innovation Foundation (approval number: DGMIF-20210721-HR-001-01) in accordance with the relevant guidelines and regulations. All participants provided written informed consent prior to 4D flow MRI experiment. Six healthy volunteers aged 22 to 33 years were prospectively recruited, and were randomly named volunteers V1–6 in this study. Electrocardiogram sensor was attached to a subject for cardiac MRI gating and respiratory gating. 4D flow MRI images were acquired for a thoracic aorta during free-breathing condition using the same clinical scanner. Venc values were selected in the range of 150‒190 cm/s to avoid aliasing of peak velocity. Cardiac number of images was 25, and the number of acquired slices were in the range of 24‒32. Scan parameters were as follows: echo time = 2.3‒3.3 ms, repetition time = 38.7‒47.2 ms, temporal resolution = 33.1‒50.48 ms, flip angle = 7‒15°, field of view = 231‒304 × 271‒384 mm^2^, voxel size = 1.7‒2.4 × 1.7‒2.4 × 1.7‒2.5 mm^3^.

### Data analysis

Magnitude and phase images acquired from 4D flow MRI were corrected through velocity anti-aliasing, noise filtering, and eddy current correction to enhance image quality. Corrected images were used to generate 3D PC MRA images. The segmentation between the fluid and non-fluid domains was conducted based on 3D PC MRA images of in vitro phantom models and in vivo aorta using ITK-SNAP software (v.3.8.0, University of Utah, Salt Lake City, UT, USA). The flow waveforms were obtained from 11 slices at the distance of 20 mm for in vitro phantom models and dozens of slices at the distance of 10 mm for in vivo aorta. The obtained flow waveforms were initially normalized using min–max values, in which the intensity values were between the minimum value of 0 and the maximum value of 1. Flow waveforms were interpolated using spline-interpolation as a function of iT. Cross-correlation algorithm was employed to calculate PTT based on the normalized and interpolated flow waveforms by comparing the upstroke region of two flow waveforms^23^.

PWV was calculated by using obtained PTT based on the Eq. (1):

1 $$\mathrm{Pulse \, wave \, velocity }(\mathrm{PWV}) (\mathrm{m} \, {s}^{-1}) = \frac{\mathrm{Distance}}{\mathrm{Pulse \, transit \, time }(\mathrm{PTT})}$$

where distance is the distance between two analysis planes based on the PC MRA image, and PTT is the time delay of the transmitted blood flow waveform relative to incident pulse waveform. First, it was assumed that PTT calculated by iT of 0.1 ms (iT_0.1 ms_) provides ‘the reference PWV’, which was previously introduced by Sonnabend et al.^27^. The flow waveforms interpolated with iT_0.1 ms_ minimize rounding errors that can be generated by discrete shift when calculating PTT. In addition, coefficient of variation (CoV) was used to optimize iT based on the Eq. (2):

2 $$\mathrm{Coefficient \, of \, variation }(\mathrm{CoV}) = \frac{\mathrm{Standard \, deviation \, of \, PTT}}{\mathrm{Mean \, of \, PTT}}$$

where the standard deviation and mean of PTT were calculated by increasing iT from iT_0.1 ms_ to iT with lower resolution. Critical iT (iT_Crit_) was subsequently defined as a specific iT when CoV exceeded certain values to simultaneously achieve minimized difference of PTT and low computation time. In this study, iT_Crit_ was calculated by CoV of 0.005. Moreover, iT of 1 ms (iT_1 ms_), which is widely used in conventional PWV measurements, was also used to compare differences of 4D flow PWV. Potential differences between the reference at iT_0.1 ms_ and the calculated PWV values at other iT were compared by employing Bland‒Altman analysis.

**Figure 2:**
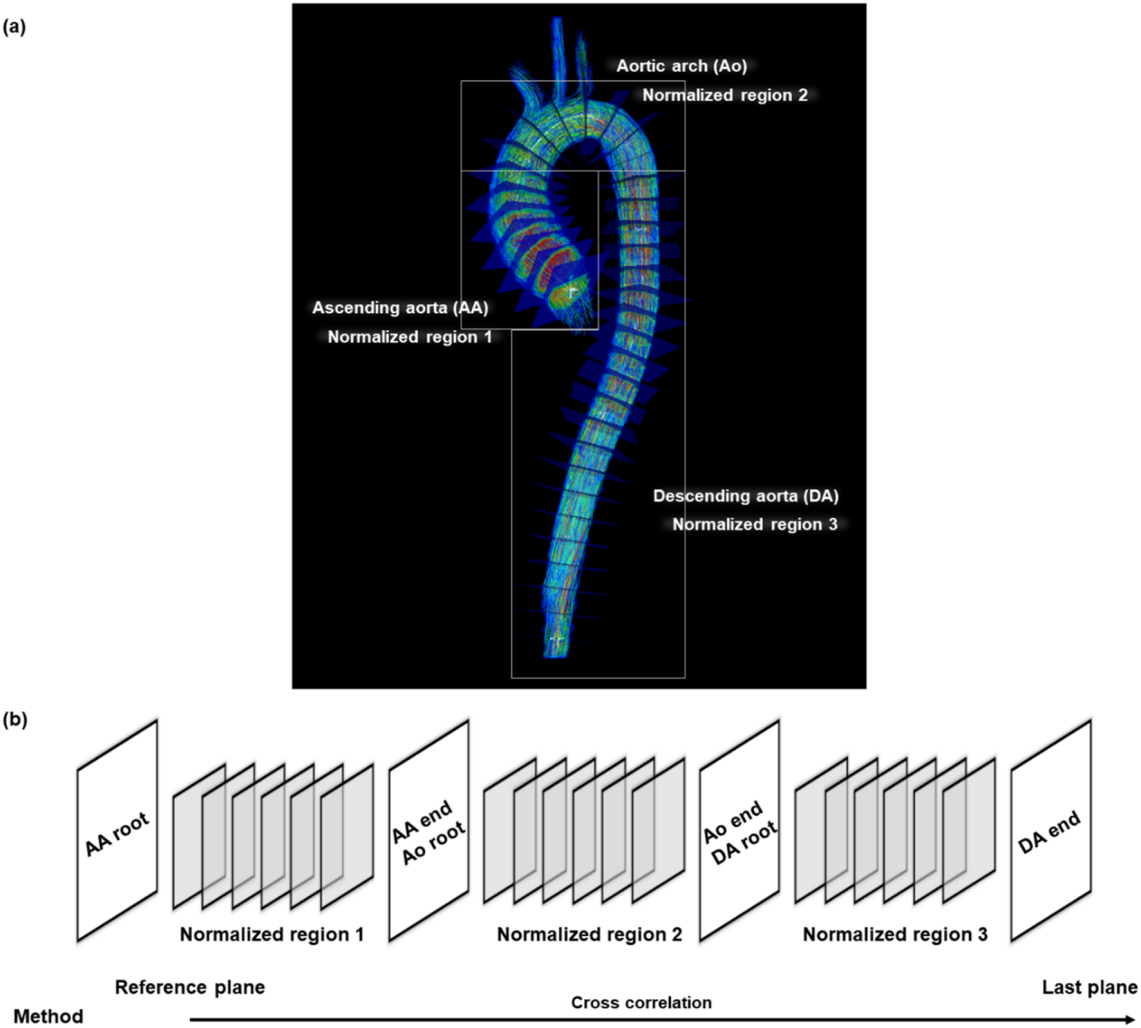
Schematics of in vivo 4D flow PWV measurement. (**a**) Segmentation of the aorta into three regions: ascending aorta (AA), aortic arch (Ao), and descending aorta (DA); these correspond to normalized regions 1, 2, and 3, respectively. (**b**) Method for calculating pulse transit time via cross-correlation by using two flow waveforms obtained from the reference plane of AA root and the other plane.

In vivo 4D flow PWV were regionally estimated at three different regions of ascending aorta (AA), aortic arch (Ao), and descending aorta (DA) (Fig. 2a). The length of each region was normalized as 0 to 1 to avoid patient-specific variations. AA, Ao, and DA were named as normalized regions 1, 2, and 3, respectively. The root plane of AA was defined as the reference plane, and PTT was calculated using two blood flow waveforms obtained from the reference plane and the other plane (Fig. 2b). For regional 4D flow PWV comparisons, iT_Crit_ values were set to be zero at AA, Ao, and DA root planes, and analyzed along aorta. Representative iT_Crit_ for all subjects was calculated as the slope of linear regression for iT_Crit_ values in each normalized region. The reference and the calculated PWV values were compared by using Bland‒Altman analysis. iT_Crit_ was further normalized by the length of in vitro phantom model or that of aorta (normalized iT_Crit_). Normalized iT_Crit_ was shown as a function of 4D flow PWV calculated by iT_Crit_ to estimate potential differences using known PWV and the used iT.

## Results

### The effect of interpolation time on in vitro 4D flow PWV measurements

**Figure 3:**
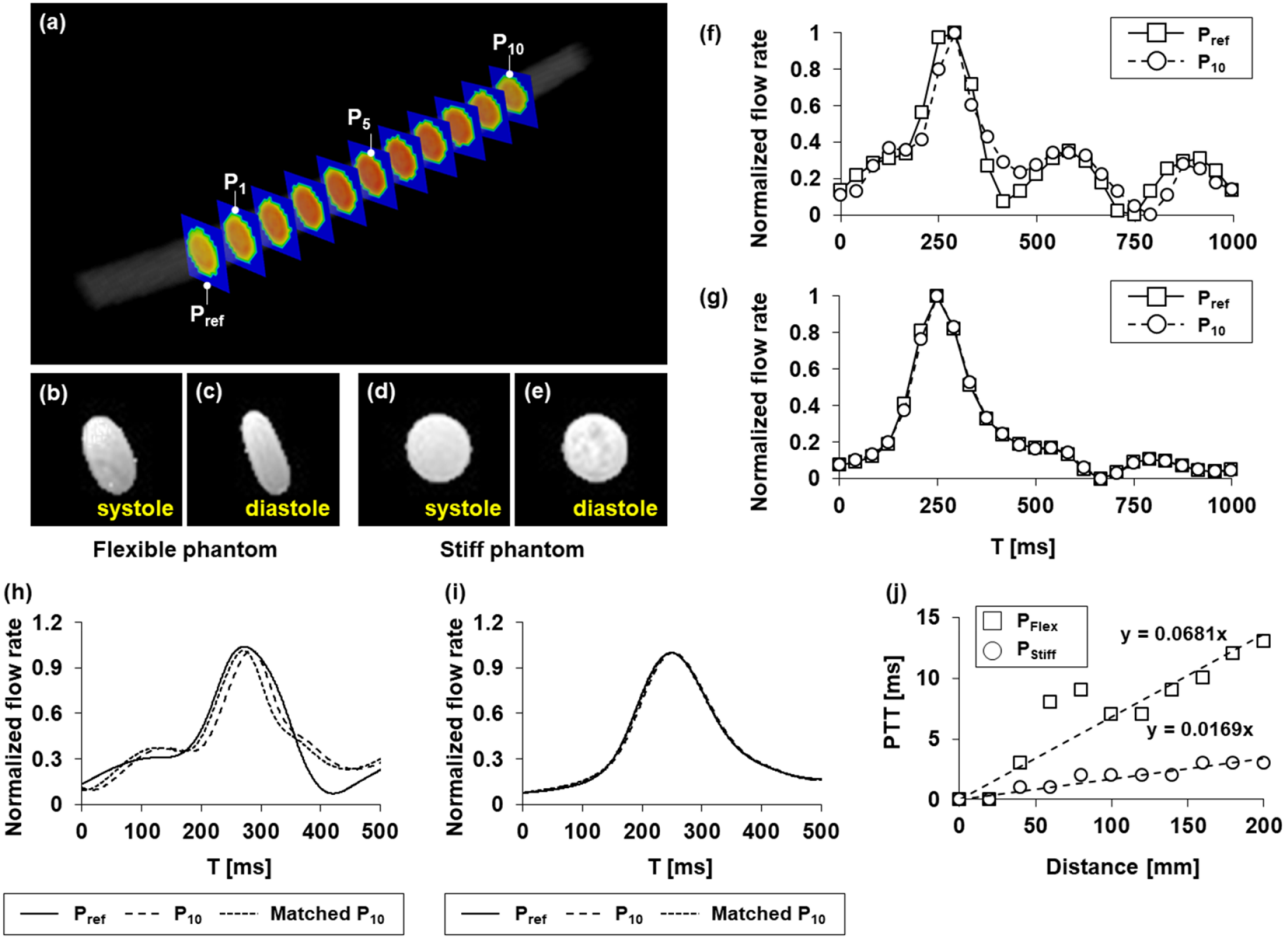
Estimation of PTT via the cross-correlation algorithm by using in vitro phantom models at the conditions of the flow rate of Q_Mid_ and the beat frequency of 60 BPM. (**a**) Analysis planes containing the reference plane (P_ref_) and 10 other planes. Morphological shapes of P_Flex_ at (**b**) systole and (**c**) diastole and P_Stiff_ at (**d**) systole and (**e**) diastole. Raw data on normalized flow waveforms at P_ref_ and 10th plane (P_10_) for (**f**) P_Flex_ and (**g**) P_Stiff_. Flow waveforms interpolated by iT_1 ms_ at P_ref_ and P_10_ and matched flow waveform via the cross-correlation algorithm for (**h**) P_Flex_ and (**i**) P_Stiff_. (**j**) Linear regression of PTT according to distance for both P_Flex_ and P_Stiff_.

4D flow PWV measurements were initially performed using an in vitro phantom model at the conditions of the flow rate of Q_Mid_ and the beat frequency of 60 BPM. 11 planes were sectioned perpendicular to a phantom model with an interval of 20 mm where the first plane was defined as the reference plane (Fig. 3a). Morphological shape of P_Flex_ is asymmetrically contracted at diastole compared to the shape at systole (Fig. 3b,c). However, the shape of P_Stiff_ is almost retained during cardiac phase (Fig. 3d,e). This result indicates that the morphological shape is significantly affected by the stiffness of phantom model. Raw data on flow waveform at 10th plane (P_10_) for P_Flex_ show the delay of PTT compared with that at the reference plane (P_ref_) (Fig. 3f). However, flow waveforms for P_Stiff_ are almost similar under the same conditions (Fig. 3g). PTT between two flow waveforms interpolated using iT_1 ms_ was subsequently quantified via cross-correlation algorithm. Interpolated waveform at P_10_ for P_Flex_ is significantly shifted to the right as the propagation time of flow waveform is delayed with PTT of 13 ms (Fig. 3h). The left-shifted P_10_ with the interval of 13 ms (matched P_10_) well corresponds to the waveform at P_ref_. On the contrary, flow waveforms at P_ref_ and P_10_ for P_Stiff_ are nearly same with PTT of 3 ms (Fig. 3i). Linear regression of PTT as a function of distance results in global PTT values of 13.62 ms and 3.38 ms for P_Flex_ and P_Stiff_, respectively (Fig. 3j), by multiplying the slope by the length of phantom model of 200 mm.

**Figure 4:**
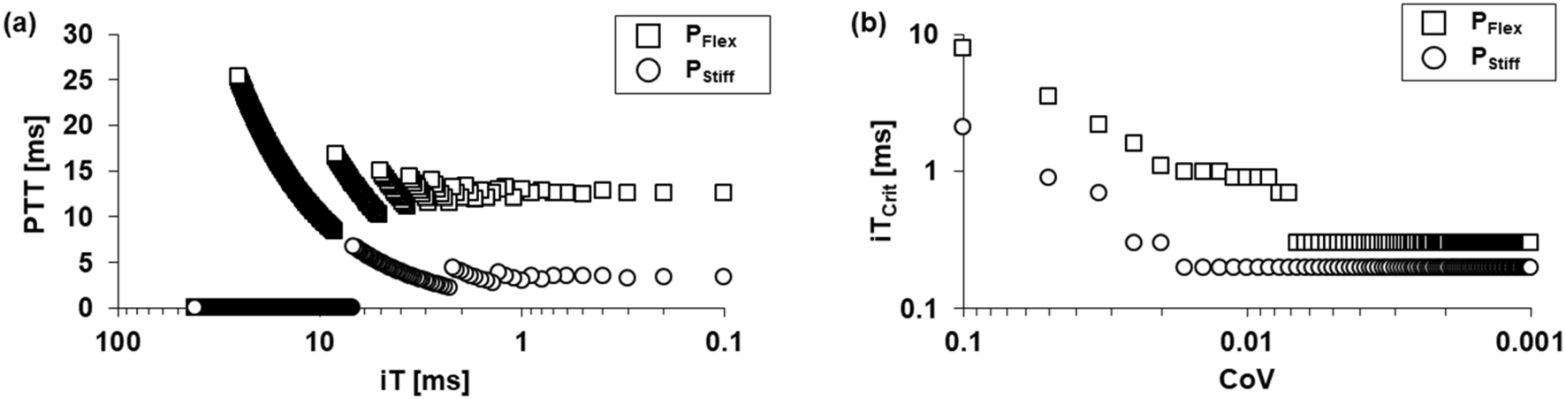
(**a**) Variation of PTT values for P_Flex_ and P_Stiff_ as a function of iT. (**b**) Variation of iT_Crit_ for P_Flex_ and P_Stiff_ as a function of CoV.

Figure 4a illustrates the variation in PTT at the conditions of Q_Mid_ and 60 BPM and at P_10_ for P_Flex_ and P_Stiff_, as a function of iT. PTT values for both phantom models exhibit a large variation when iT values increase to a lower temporal resolution. As iT values decrease to a higher resolution, PTT values begin to reach an equilibrium. The equilibrium PTT values calculated by iT_0.1 ms_ were 12.6 ms and 3.4 ms for P_Flex_ and P_Stiff_, respectively. Especially, P_Stiff_ has lower PTT values compared to PTT for P_Flex_, because flow waveforms are almost similar. This indicates that a stiff vessel would have a large variation of PTT by small changes of iT. To analyze the deviation of PTT based on that at equilibrium, iT_Crit_ was shown as a function of CoV (Fig. 4b). For P_Flex_, iT_Crit_ is 0.3 ms as CoV reaches to 0.006 or smaller value. This result implies that the deviation of PTT will be comparably small when CoV sets to 0.006, similar to the deviation at equilibrium. However, when CoV sets to a larger value, the potential difference of PTT, calculated at a larger iT than 0.3 ms, increases compared to the deviation at equilibrium.

On the contrary, P_Stiff_ requires iT_Crit_ of 0.2 ms even at CoV of 0.02, while iT_Crit_ for P_Flex_ is 1.1 ms at CoV of 0.02. This means that P_Stiff_ requires higher temporal resolution compared to P_Flex_ to achieve a comparably small difference compared to PTT at equilibrium. Adopting low CoV allows for accurate PTT measurement, while requiring a long computation time. In this study, we adopted CoV of 0.005 to simultaneously reduce a potential difference and a computation time in 4D flow PWV measurement.

4D flow PWV values according to flow rates and iT for P_Flex_ and P_Stiff_ were shown in Table 3. The result indicates that PWV values are non-linearly varied for both P_Flex_ and P_Stiff_. P_Flex_ has the largest PWV value obtained at Q_Low_, while the lowest PWV value is obtained at Q_Mid_. On the contrary, P_Stiff_ has the largest PWV value obtained at Q_Mid_ and the lowest PWV value obtained at Q_High_. This might be attributed to the variation in absorbed energy to silicone tubes during systole according to the flow rates and stiffness of phantom models. The effect of beat frequency and iT on the calculation of PWV values was shown in Table 4. PWV values are also largely varied for both P_Flex_ and P_Stiff_. However, PWV values tend to decrease as beat frequency increases. This tendency might be attributed to the difference in the formation of flow waveforms according to Wo numbers.

**Table 3:** In vitro PWV values obtained from iT_0.1 ms_, iT_1 ms_, and iT_Crit_ according to flow rates.

PWV (m s^−1^)						
Flow rate	P_Flex_			P_Stiff_		
	iT_0.1 ms_	iT_1 ms_	iT_Crit_	iT_0.1 ms_	iT_1 ms_	iT_Crit_
Q_Low_	18.47	18.78	18.47	52.70	51.33	52.70
Q_Mid_	14.68	14.69	14.68	62.81	59.23	61.80
Q_High_	17.10	17.15	17.10	44.08	41.85	44.05

**Table 4:** In vitro PWV values obtained from iT_0.1 ms_, iT_1 ms_, and iT_Crit_ according to beat frequency.

PWV (m s^−1^)						
Beat frequency (BPM)	P_Flex_			P_Stiff_		
	iT_0.1 ms_	iT_1 ms_	iT_Crit_	iT_0.1 ms_	iT_1 ms_	iT_Crit_
60	14.68	14.69	14.68	62.81	59.23	61.80
80	5.51	5.55	5.54	26.63	26.64	26.72
100	9.19	9.23	9.17	21.70	21.94	21.68
120	4.54	4.55	4.54	26.12	25.93	26.12

**Figure 5:**
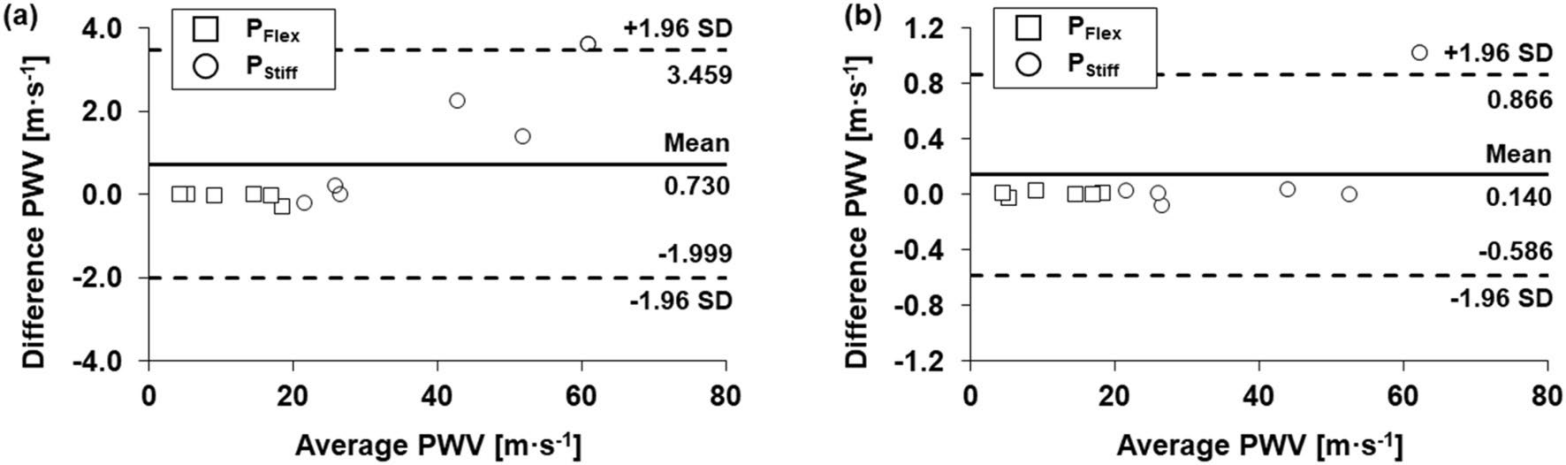
Bland‒Altman plots of (**a**) PWV values obtained from iT_0.1 ms_ and iT_1 ms_ and (**b**) PWV values obtained from iT_0.1 ms_ and iT_Crit_ for both P_Flex_ and P_Stiff_.

Potential differences were estimated by employing Bland‒Altman analysis between the reference PWV and PWV calculated by iT_1 ms_ or iT_Crit_. Mean difference of PWV calculated by iT_1 ms_ is 0.730 m s^−1^ and the 95% limitation agreement is ‒ 2.0 to 3.5 m s^−1^ (Fig. 5a). However, potential difference of PWV calculated by iT_Crit_ significantly decreases more than 5 times with mean difference of 0.140 m s^−1^ and 95% limitation agreement of − 0.59 to 0.87 m s^−1^ (Fig. 5b). This result suggests that the use of iT_Crit_ in PWV measurements can reduce potential differences, compared to PWV values at equilibrium.

### Optimization of in vivo 4D flow PWV measurements

**Figure 6:**
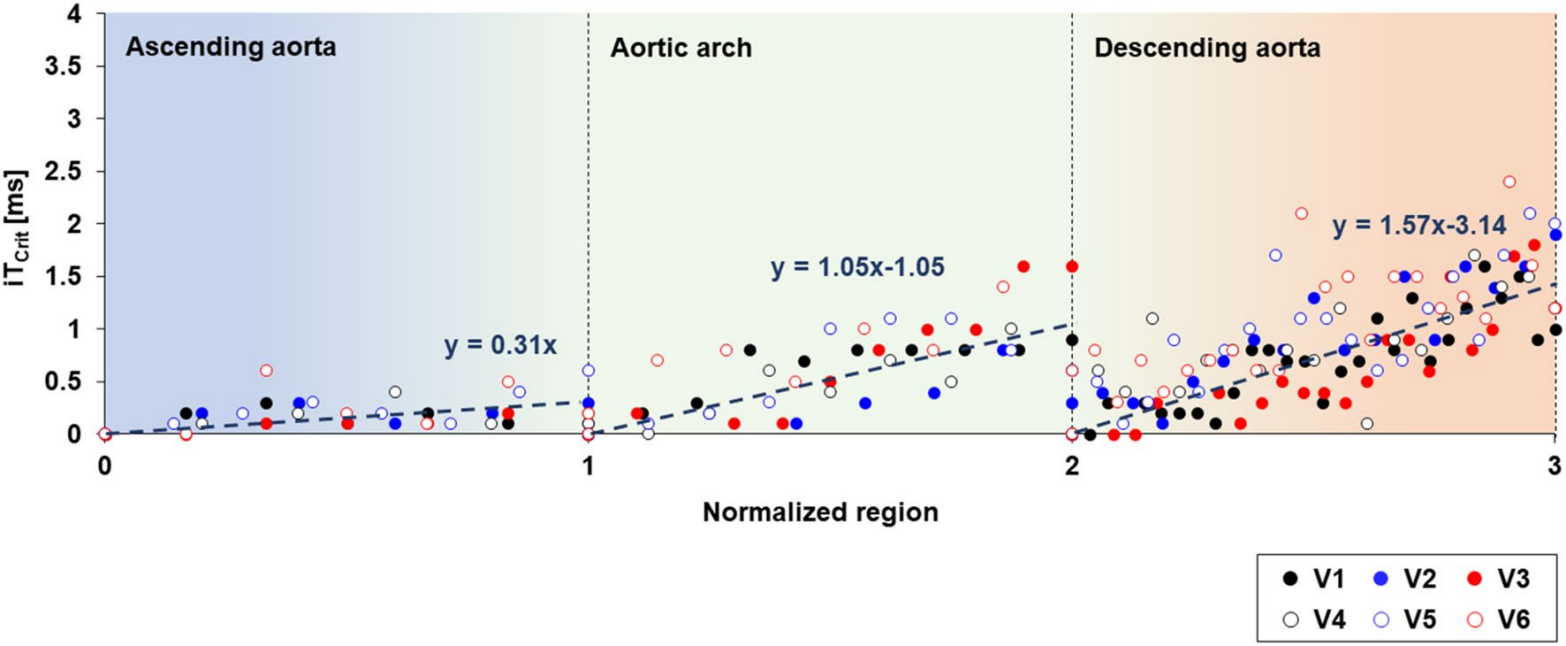
iT_Crit_ calculated by using CoV of 0.005 along aorta for all subjects where the slope of each normalized region corresponds to mean iT_Crit_.

The clinical applicability of iT_Crit_ for in vivo 4D flow PWV measurements was evaluated in six healthy subjects. Representative iT_Crit_ values of 0.3, 1.1 and 1.6 ms were initially obtained from the slope of linear regression at the normalized regions 1, 2, and 3 for all subjects, respectively (Fig. 6). PWV values calculated by iT_0.1 ms_, iT_1 ms_, and iT_Crit_ were summarized in Table 5. A higher resolution iT_Crit_ in the region of AA is required compared to other regions, because flow waveform rapidly propagates with a small PTT. This result is well consistent with the result of in vitro PWV for P_stiff_ (Fig. 4). This result also implies that PWV calculated by iT_1 ms_ in the region of AA may possess a large potential difference compared to PWV at equilibrium. In contrast, iT_Crit_ in the region of DA relatively increases compared to iT_1 ms_, because two flow waveforms are separated enough to distinguish time delay using a low resolution iT. This also suggests that using iT_Crit_ for PWV measurements reduces computation time (Supplementary Fig. 1), which will be helpful for evaluating 4D flow PWV in large cohort studies compared to that by using iT_1 ms_, while also minimizing potential differences in PWV values.

**Table 5:** In vivo PWV values obtained from iT_0.1 ms_, iT_1 ms_, and iT_Crit_ according to the region of aorta in six healthy volunteers.

PWV (m s^−1^)									
Volunteer	AA			Ao			DA		
	iT_0.1 ms_	iT_1 ms_	iT_Crit_	iT_0.1 ms_	iT_1 ms_	iT_Crit_	iT_0.1 ms_	iT_1 ms_	iT_Crit_
V1	6.94	6.84	6.85	3.09	3.06	3.16	6.04	6.03	6.07
V2	4.86	4.95	4.84	2.21	2.18	2.22	3.67	3.67	3.65
V3	6.20	6.23	6.22	1.84	1.85	1.86	4.67	4.69	4.67
V4	4.46	4.51	4.47	2.42	2.43	2.45	5.58	5.63	5.54
V5	4.62	4.58	4.59	2.34	2.33	2.31	4.22	4.25	4.19
V6	3.62	3.63	3.63	2.68	2.69	2.72	4.70	4.69	4.70

**Figure 7:**
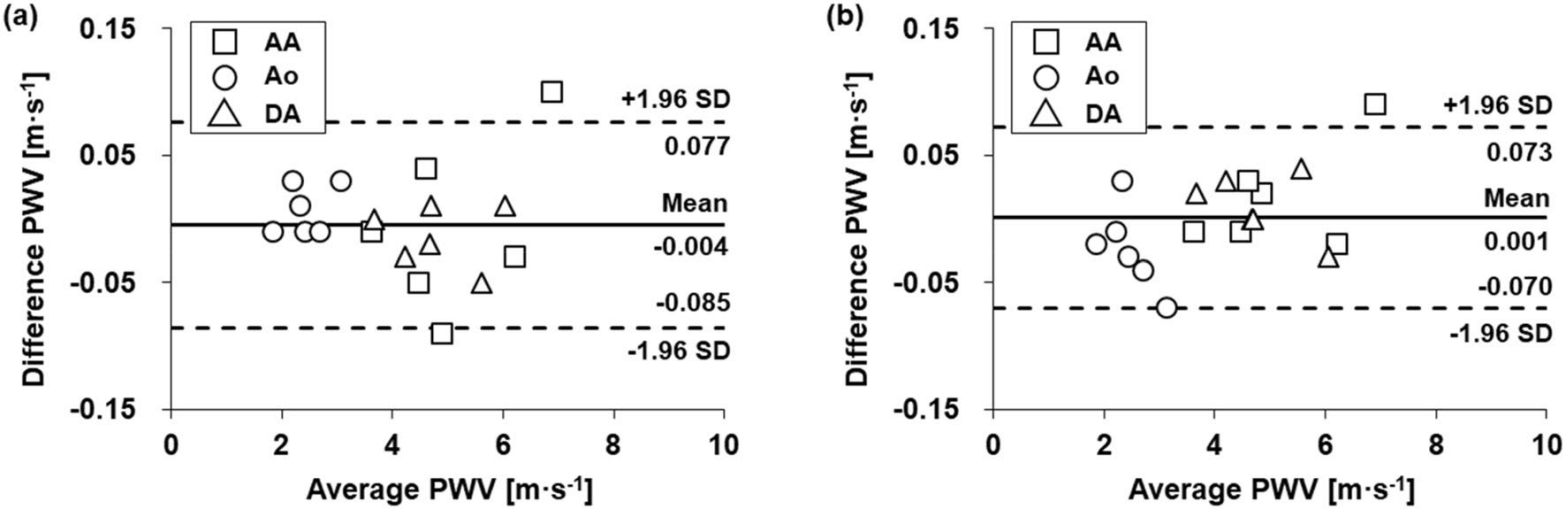
Bland‒Altman plots of (**a**) PWV values obtained from iT_0.1 ms_ and iT_1 ms_ and (**b**) PWV values obtained from iT_0.1 ms_ and iT_Crit_ for six healthy volunteers.

Bland‒Altman analysis was further employed to evaluate mean difference in PWV values in healthy subjects. Mean difference between PWV values obtained at iT_0.1 ms_ and iT_1 ms_ is ‒ 0.004 m s^−1^ and the 95% limitation agreement is ‒ 0.09 to 0.08 m s^−1^ (Fig. 7a). On the contrary, mean difference between PWV values obtained at iT_0.1 ms_ and iT_Crit_ is 0.001 m s^−1^ and the 95% limitation agreement is − 0.07 to 0.07 m s^−1^ (Fig. 7b). Potential differences of PWV calculated by iT_1 ms_ tend to increase as mean PWV values increase. However, potential differences of PWV calculated by iT_Crit_ seem to be sparsely distributed regardless of mean PWV values. This tendency is also observed in Bland‒Altman analysis for in vitro 4D flow PWV results (Fig. 5). This result demonstrates that PWV values, differently obtained at iT_Crit_ of 0.3, 1.1 and 1.6 at AA, Ao, and DA, respectively, have low potential differences compared to PWV values obtained at iT_1 ms_.

**Figure 8:**
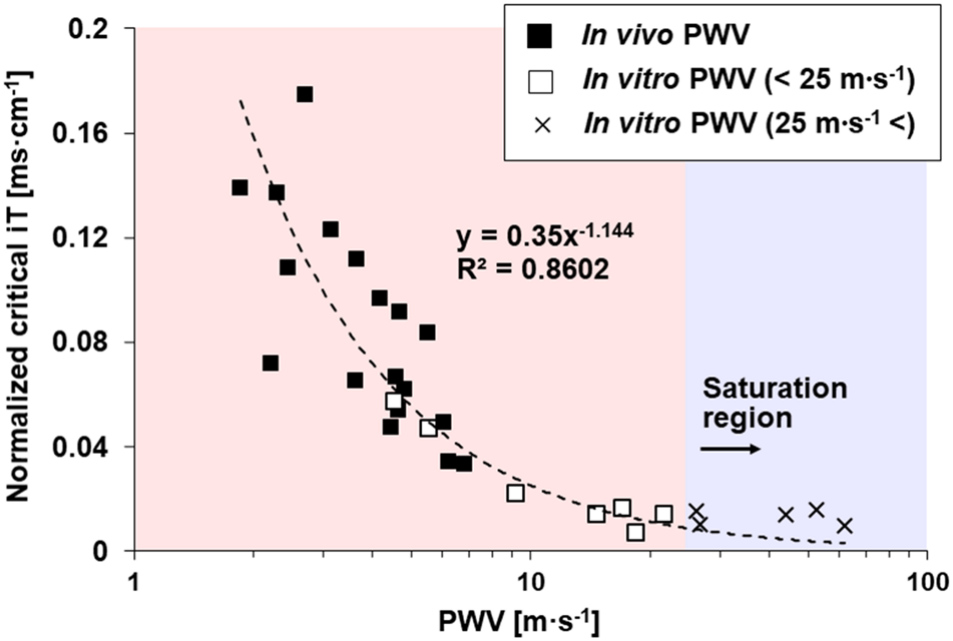
iT_Crit_ normalized by distance according to in vivo and in vitro PWV values.

Based on in vitro and in vivo experiments, a novel equation was proposed by using iT_Crit_ normalized by distance between two analysis planes (normalized iT_Crit_), as a function of PWV calculated by iT_Crit_ (Fig. 8). Below the PWV value of 25 m s^−1^, normalized iT_Crit_ is well fitted by the equation of $0.35{x}^{-1.144}$ with an R^2^ of 0.8602. For example, subject V1 has PWV of 6.84 m s^−1^ in the AA region with a length of 6 cm. We can calculate the corresponding normalized iT_Crit_ in the region of AA as 0.039 ms cm^−1^. Then, iT_Crit_ can be deduced approximately as 0.2 ms when multiplying normalized iT_Crit_ by the length. This result suggests using iT_Crit_ of 0.2 ms for PWV measurement, instead of iT_1 ms_, to minimize potential difference from PWV values at equilibrium. On the contrary, subject V2 has PWV value of 3.67 m s^−1^ in the DA region with a length of 16 cm. The corresponding normalized iT_Crit_ in the region of DA is calculated as 0.079 ms cm^−1^. Then, iT_Crit_ can be deduced approximately as 1.3 ms. This result indicates that computation time can be reduced by using iT_Crit_ while maintaining low potential difference. In contrast, above the PWV value of 25 m s^−1^, normalized iT_Crit_ seems to reach an equilibrium. This implies that iT_0.1 ms_ is essentially required to obtain PWV with low difference, particularly in high PWV regimes.

## Discussion

When considering aortic PWV change of 14.8% corresponding to 10 years of arterial aging^28^, potential differences of 0‒2% and 0‒6%, which are obtained from in vivo and in vitro PWV estimations, respectively, may induce serious drawbacks in stiffness estimation. The aims of this study were to identify the potential differences, as the shape of flow waveforms varies during 4D flow PWV measurements. Proposed iT_Crit_ reduces the potential difference in PWV measurements.

PWV is known to be positively correlated with age and mean blood pressure (MBP) levels. PWV for healthy volunteers are in the range of 6–11 m s^−1^^29^. Younger healthy volunteers exhibit mean PWV of 6.2 m s^−1^, while mean PWV of 10.9 m s^−1^ is typical for elderly healthy volunteers. The results of 4D flow PWV measurements in our study indicate that PWV values obtained from the P_Flex_ are in the range of 4‒19 m s^−1^ (Figs. 3, 4), and a part of them well corresponds with PWV values of healthy volunteers. PWV values generally increases with increasing MBP in all age categories^29^. In addition, elevated heart rate normally increases PWV values for rats and humans under conditions of consistent mean arterial pressure and blood pressure levels^30,31^. However, we need to acknowledge that PWV values obtained in this study are not well correlated with such a tendency. This might be attributed to the mismatch of Re or Wo numbers compared to those observed in human, thereby deviating the propagation of flow waveforms with different energy absorption and pressure distribution^32^. Therefore, it is required to consider simultaneously Re and Wo numbers to further investigate the effect of hemodynamic conditions on the estimation of PWV values in more details.

Our results suggest that the magnitude of PWV values is closely correlated with iT_Crit_, where a high resolution iT_Crit_ is required to calculate a high PWV value with low difference. This is attributed to the rapid propagation of flow waveforms with a very small PTT between two waveforms. In this way, traditional iT_1 ms_ induces a difference of approximately 2%, corresponding to PWV value of 18.47 m s^−1^. Houriez et al.^22^ assessed three different algorithms and three strategies in 4D flow PWV measurement by interpolating flow waveforms with iT_1 ms_. Elderly volunteers have significantly higher PWV values in the range of approximately 7‒14 m s^−1^, compared with PWV values of younger volunteers in the range of approximately 5‒8 m s^−1^. Based on our results, these PWV values might have potential differences in each algorithm especially for elderly volunteers. However, it should be noted that these differences are relatively smaller than the changes in PWV values between younger and elderly volunteers (40%). Similarly, MRI-based PWV measurements were also validated with two external devices, a Complior II (Artec-Medical) and a PulsePen (DiaTecne), using iT_1 ms_^33^. However, the potential differences in PWV measurements might still occur, particularly at higher PWV values. Thus, careful consideration in selecting iT would be essential to minimize a potential difference, especially in a patient with high PWV values.

In contrast, some PWV values obtained from P_Stiff_ are significantly outside the normal range (Tables 3 and 4). The tendency of PWV changes between P_Flex_ and P_Stiff_ is also different. A stiffer phantom model will have faster transmission of the waveforms, inducing smaller PTT values. The higher the PWV values, the larger the potential differences that would be generated. Thus, stiffer phantom models require a small iT to obtain PWV values with low potential difference. For an example, patients who suffer from aortic aneurysm have increased PWV values in the range of approximately 17‒19 m s^−1^^34^. In addition, aortic arch replacement by prosthetic graft replacement, frozen elephant trunk technique, and hybrid surgery induces a significant increase in PWV values to 19‒23 m s^−1^. In this range of PWV values, the use of an interpolation time of 1 ms would induce large potential differences in PWV measurements. The proposed iT_Crit_ in this study can provide PWV values with a comparably low potential difference, thereby providing an effective method for matching PWV and aortic compliance before and after surgery to reduce the risk of cardiovascular diseases.

The stiffness of the aorta increases in the order of AA, Ao, and DA, as revealed by computation modeling of age-related aortic stiffness changes with human hemodynamics^35^. In contrast, the tendency of PWV values obtained from computation modeling exhibits a low correlation with the circumferential stiffness. This is attributed to non-normalized arterial geometry with different radii and wall thicknesses. Our results also show that in vivo PWV values in the AA region are initially higher than those at Ao with a low correlation related to aortic stiffness, as previously reported. Based on the Moens-Korteweg equation, when assuming a constant wall thickness, the radius of the vessel is inversely proportional to the square of the PWV values. Theoretically, a large radius of the AA region can offset a high PWV value in the estimation of aortic stiffness^36^. Thus, in vivo PWV values in the AA region with a large diameter would have low regional stiffness, even though PWV values are high.

To minimize potential differences, Guala et al.^21^ used 100 analysis planes in the AA and DA regions, instead of using a high resolution iT. Harloff et al.^16^ also evaluated aortic stiffness based on 50‒60 analysis planes using three different algorithms (TTF, the 50%-rule that calculates the time at which the flow rate becomes half of the peak flow rate, and cross-correlation) for compensating the low temporal resolution of 20 ms in 4D flow MRI, compared with the high temporal resolution of approximately 10 ms in 2D PC MRI. A large number of planes certainly gives a small difference in PWV measurements, but it increases computation time. In addition, Houriez et al.^22^ also investigated the differences of PWV values according to 20 and 50 frames per cardiac cycle with an interpolation time of 1 ms based on TTF, cross-correlation, and Fourier transforms. They demonstrated the robustness of the wavelet-based method with 20- and 50-phase reconstruction, which is in strong agreement with age, even in volunteers aged 50 years or older.

Although such 4D flow MRI techniques are well established, this study newly suggests that the existence of potential differences should be considered based on the various parameters affecting 4D flow PWV measurements. In general, the interpolation method is applied to already measured flow waveforms using 4D flow MRI technique with approximately 40 ms temporal resolution. To acquire missing time steps in flow waveforms, many researchers create flow or pressure information at unmeasurable time point by interpolating waveforms. It is noteworthy that the ideal approach is to acquire real information at missing time steps using a measurement technique with a high temporal resolution. However, when faced with limitations that cannot improve temporal resolution of measurement techniques, interpolation becomes an alternative way to estimate information at those steps. Our study also suggests that temporal resolution of measurement techniques may play an important role in PWV measurements with low potential differences, given that the shape of flow waveforms will vary as a function of measurement techniques.

This study newly provides a guideline for minimizing the potential differences with achieving low computation time. The proposed equation of 4D flow PWV vs. normalized iT may be further utilized to estimate the potential differences in 4D flow PWV regardless of patients' group, as well as normative subjects. In addition, this study might be helpful for understanding potential differences in 4D flow PWV measures, as a function of flow propagation speed, which may occur in traditional PWV measurements.

## Conclusion

This study provides 4D flow PWV with low potential differences and low computation time, achieved by using iT_Crit_ through in vitro and in vivo 4D flow PWV measurements. In addition, the difference estimation model with normalized iT_Crit_ according to the 4D flow PWV values is introduced, ensuring good alignment of all in vitro and in vivo data. Therefore, the present study would hold the potential to identify the subtle changes in aortic stiffness for patients with cardiovascular disease using 4D flow MRI technique for the clinical purposes.

### Supplementary Information

Supplementary Figure 1.

## Data Availability

The datasets used and/or analyzed during the current study are available from the corresponding author upon reasonable request.
