# Supplementary material for: Interpolation time-optimized aortic pulse wave velocity estimation by 4D flow MRI
Source: Sci Rep. 2023 Sep 30;13:16484. doi: 10.1038/s41598-023-43799-z (PMC10542805; doi:10.1038/s41598-023-43799-z)
Supplement: Supplementary file 1 — Supplementary Figure 1. [file 41598_2023_43799_MOESM1_ESM.docx]

**Supplementary Figure 1**


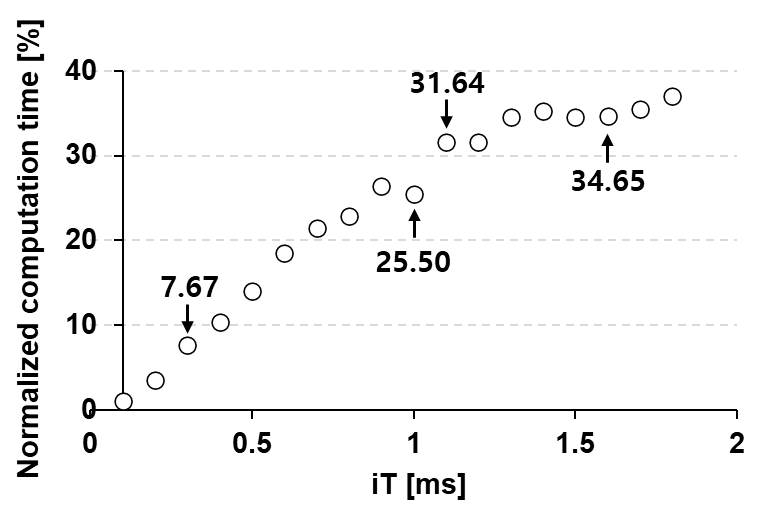


**Fig. S1** Normalized computation time as a function of iT. Representative iT_Crit_ at AA, Ao, and DA, and iT_1ms_ values were shown with arrows. The computation time at iT_Crit_ in AA is 3.3 times slower than the computation time at iT_1ms_, while reducing potential differences. On the contrary, the computation time at iT_Crit_ in DA is 1.4 times faster than the computation time at iT_1ms_.
